# Long‐term spaceflight composite stress induces depressive behaviors in model rats through disrupting hippocampus synaptic plasticity

**DOI:** 10.1111/cns.14438

**Published:** 2023-10-17

**Authors:** Yi‐Shu Yin, Yuan‐Bing Zhu, Jun‐Lian Liu, Quan‐Chun Fan, Xiao‐Rui Wu, Shuang Zhao, Jia‐Ping Wang, Yu Liu, Yong‐Zhi Li, Wei‐Hong Lu

**Affiliations:** ^1^ School of Chemistry and Chemical Engineering Harbin Institute of Technology Harbin China; ^2^ School of Medicine and Health Harbin Institute of Technology Harbin China; ^3^ National and Local Joint Engineering Laboratory for Synthesis, Transformation and Separation of Extreme Environmental Nutrients Harbin China; ^4^ China Astronaut Research and Training Center Beijing China; ^5^ The Intelligent Equipment Research Center for the Exploitation of Characteristic Food & Medicine Resources, Chongqing Research Institute, Harbin Institute of Technology Chongqing China

**Keywords:** depression, long‐term spaceflight composite stress, LTP, NMDAR channel, synaptic plasticity

## Abstract

**Introduction:**

Long‐term spaceflight composite stress (LSCS) can cause adverse effects on human systems, including the central nervous system, which could trigger anxiety and depression.

**Aims:**

This study aimed to identify changes in hippocampus synaptic plasticity under LSCS.

**Methods:**

The present study simulated the real long‐term space station environment by conducting a 42‐day experiment that involved simulating microgravity, isolation, noise, circadian rhythm disruptions, and low pressure. The mood and behavior of the rats were assessed by behavior test. Transmission electron microscopy and patch‐clamp were used to detect the changes in synapse morphology and electrophysiology, and finally, the expression of NMDA receptor channel proteins was detected by western blotting.

**Results:**

The results showed that significant weight loss, anxiety, and depressive behaviors in rats were observed after being exposed to LSCS environment for 42 days. The synaptic structure was severely damaged, manifested as an obvious decrease in postsynaptic density thickness and synaptic interface curvature (*p* < 0.05; *p* < 0.05, respectively). Meanwhile, LTP was significantly impaired (*p* < 0.0001), and currents in the NMDAR channel were also significantly reduced (*p* < 0.0001). Further analysis found that LSCS decreased the expression of two key subtype proteins on this channel.

**Conclusion:**

These results suggested that LSCS‐induced depressive behaviors by impairing synaptic plasticity in rat hippocampus.

## Graphical abstract

LSCS‐induced depressive behaviors by impairing synaptic plasticity in rat hippocampus. The underlying mechanism may be that LSCS lead the failure of NMDAR channels to open normally by reducing the expression of key subtypes NR2A and NR2B, resulting in the dysregulation of intracellular calcium levels so that LTP cannot be triggered normally, and finally lead the impairment of synaptic plasticity.

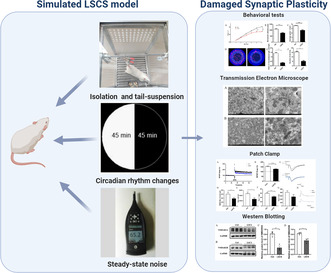

## 1 INTRODUCTION

In the future, manned spaceflights will head toward the Moon and deep space, which means that astronauts need execute space missions for a much longer period, approximately several months to years.^1^ Whether the space mission can be successfully completed depends not only on the precision equipment of the spacecraft but also on the normal and healthy operation status of the astronauts. Mounting research indicates that the space environment can cause damage to human body, including cardiovascular dysfunction, muscle atrophy and bone loss, and so on.^2^, ^3^ Moreover, the space environment also have adverse impact on the central nervous system (CNS).^4^ Some studies have reported that after performing missions of long duration, astronauts experienced significant changes in brain structure such as upward shift of the brain and narrowing of the central sulcus and the cerebrospinal fluid spaces at the vertex.^5^ Evidence displayed that it would cause depression, anxiety, and cognitive impairment as well, which greatly limit the time that human can spend in space.^6^ However, the impact of the space environment on the CNS is largely unknown, and still no clear explanation for the cause of depression and anxiety.

One crucial building region in human brain that related to depression, anxiety, and cognitive is hippocampus, which may attribute to its highly plastic and stress‐sensitive.^7^ In previously reported studies, we established that Long‐term spaceflight composite stress (LSCS) could induce depression‐like behavior and neuronal damage in hippocampus of rats.^8^ Further, it has been proposed that the pathogenesis of depression is closely related to hippocampus synaptic plasticity.^9^ Certain research shows hippocampus changes to synaptic form and function occur in depression.^10^ Reports on major depressive disorder patients also provides evidence in support of a reduction of synapse number, including decreased levels of glutamate receptor subtypes, presynaptic neurotransmitter vesicle‐associated proteins, and the postsynaptic functional proteus hippocampus, and other forebrain structures.^11^, ^12^, ^13^ Based on these evidences, we, therefore, come up with our hypothesis that the possible underlying mechanism of LSCS‐inducing depression could lie in its damage on synaptic plasticity.

In this study, we used a combination of five factors to simulate the LSCS environment, including microgravity, isolation and confinement, noise, circadian rhythm disruptions, and low pressure, which were much closer to the real space station environment than simply simulating a single factor. We examined body weight, depressive behaviors, and synaptic morphology in the LSCS environment. We also looked for alterations in electrophysiology, including LTP, one of the major forms of synaptic plasticity changes, and NMDAR channel currents, which is the key calcium channel responsible for LTP.^14^ Thus, we hoped to elucidate the underlying mechanisms of altered synaptic plasticity under LSCS. To the best of our knowledge, this is the first report to simulate real space station environment through five factors as well as exploring potential molecular mechanism of depression in spaceflight from the perspective of synaptic plasticity, which could help to inform a more targeted approach to treating these spaceflight stress‐induced disorders.

## 2 MATERIALS AND METHODS

### 2.1 Animals

Healthy 6‐week‐old male Sprague–Dawley (SD) rats with body weight of 200 ± 10 g were purchased from Beijing Weitong Lihua Laboratory Animal Co., Ltd. (Beijing, China) and were reared in the animal room with temperature maintained at 23 ± 2°C and 12 h:12 h light–dark cycle. The animals were allowed to acclimatize to the environment for 7 days and had free access to food and water during the whole experiment. Animal raising and experiments were carried out in accordance with the recommendations of the guidelines for the use and care of live animals approved by the Animal Care and Use Committee of China Astronaut Research and Training Center. A total of 20 rats were used in this study, randomly subdivided into the control group (Ctrl, *n* = 10) and the LSCS group (LSCS, *n* = 10).

### 2.2 Construction of the LSCS model

The LSCS model was constructed with five conditions, including tail suspension, isolation rearing, steady‐state noise, circadian rhythm disruptions, and low pressure. The first four factors have been described in our previous studies,^8^ and the additional one is implemented by the low‐pressure cabin which was set to 0.9 standard atmospheres. Briefly, each model rat was tail‐suspended in an individual cage with ground glass, exposing to 65 ± 2 dBA steady‐state noise 12 h a day in the low‐pressure cabin with temperature maintained at 23 ± 2°C and 45 min:45 min light–dark cycle. The rats in the control group were reared five per cage with a natural light–dark cycle (12‐h light and 12‐h dark), while the model rats were reared in the LSCS environment. The model construction was maintained for 42 days until subsequent experiments.

### 2.3 Open field test

The open field test was carried out on Day 43. The experimental device was a black circular open box with a diameter of 80 cm and a height of 30 cm. After being placed gently in the center of the open box, the activity of the rat was recorded via a video recorder for 5 min. The experimental software automatically recorded the movement distance and calculated the average speed. After the test of each animal, the feces were removed, and the inner wall and bottom of the open box were sprayed with 70% ethanol so as to prevent the residual odor of the previous animal from affecting the next animal. Statistical analysis was performed on the movement distance and average speed.

### 2.4 Light–dark box shuttle test

The light–dark box shuttle test was carried out on Day 44. The experimental device was a cuboid of 70 × 25 × 45 cm, which consisted of a light box and a dark box of equal size. The light box was equipped with a light source, while the dark box was completely black. A circular arch was set at the connection between the two boxes for animals to pass through. When the test began, each rat was placed in the arch, and the behavioral changes were recorded by the video recorder for 5 min, including the residence time of the two boxes and the number of shuttles. After the end of each test, the feces were removed, and the bottom of the box was sprayed with 70% ethanol. The time ratio of light and dark boxes was calculated by the residence time in the light box / that in the dark box. Statistical analysis was performed on the time ratio and the times of shuttle.

### 2.5 Transmission electron microscope

After the behavioral tests, the rats were deeply anesthetized and perfused with 0.9% saline and 4% paraformaldehyde in turn. Brains were quickly removed on ice, the hippocampus was dissociated, cut into 1 mm^3^ pieces, and fixed in 2.5% glutaraldehyde overnight followed by fixing in 1% osmic acid for 2 h. The samples were then dehydrated with acetone and sectioned into 70 nm pieces. Finally, the sections were stained with 3% uranyl acetate and 0.5% lead citrate for 15 min and observed under the transmission electron microscopy (TEM). Synaptic interface parameters, including length of synaptic active area, postsynaptic density (PSD) thickness, and synaptic interface curvature, were all analyzed with Image‐Pro Plus 6.0 image analysis software.

### 2.6 Slice preparation for patch‐clamp

Acute hippocampus slices were prepared from each group. The rats were deeply anesthetized, and sacrificed by decapitation. The brains were quickly removed and transferred to ice‐cold artificial cerebrospinal fluid (ACSF) containing 185 Sucrose, 2.5 KCl, 1.2 NaH_2_PO_4_.2H_2_O, 25 NaHCO_3_, 25 D‐Glucose, 0.5 CaCl_2_, and 10 MgSO_4_ saturated with 95% O_2_ and 5% CO_2_ to pH 7.3 (in mM). The right hippocampus was then dissected and fixed on an agar block to keep it upright. The hippocampus tissue was transected with a vibratome (Leica, VT 1000 S, Germany) to a thickness of 380 μm. During the whole procedure, the slices were continuously carbonated with 95% O_2_ and 5% CO_2_, which were then transferred to an incubation tank containing the same ACSF, and incubated at room temperature for 1 h prior to electrophysiological recording.

### 2.7 Recording of LTP in hippocampus CA3‐CA1 region

The recorded LTP belonged to the Schaffer collateral pathway in hippocampus CA3‐CA1 region. The extracellular recording electrodes were placed in the stratum radiatum of the CA1 region, the stimulation electrodes were placed in the stratum radiatum of the CA3 region, and the stimulating distance between which was 400 μm. Field excitatory postsynaptic potentials (fEPSPs) were excited by stimulating electrodes placed in the CA3 area. Run a single test pulse of 100 μs duration with stimulation parameters from 10 to 40 μA in I = 0 mode. The slope of fEPSP was expressed as the slope at 20%–80% of the straight line fitted from the base of the presynaptic fiber peak to the peak of EPSP. Measure the stimulus–response curve at the beginning of each experiment, adjust the stimulus intensity to find the maximum value, take the stimulus intensity that can cause 40%–50% of the maximum response amplitude as the basic stimulus intensity, and record as the baseline that was stable more than 30 min after the response and the fluctuation range of the slope less than 10%. Basal stimulation was run immediately after high‐frequency stimulation (HFS) (100 Hz 1 s, three times with 10 s interval between each) to induce LTP, followed by stimulation with a single test pulse for 60 min. Data were digitized at 20 kHz and analyzed with pClamp10.5 software.

### 2.8 Recording of NMDA receptor channel currents

When the membrane potential was clamped at +50 mV, stimulating electrodes induced outward currents in neurons in the CA1 region of the hippocampus. Add 10 μM GABA receptor antagonist Bicuculline in the perfusate to block the GABA current, and then 20 μM AMPA receptor antagonist DNQX was added to block the AMPA current, thereby obtaining pure NMDAR current. At this time, the membrane potential was clamped at +50 mV, and a suitable stimulation intensity was given until the NMDAR current reached about 100 pA. Then 10 traces were recorded, and the interval between each trace was 30 s. Finally, D‐AP5 was added to inhibit the NMDAR current. Changes in series resistance before and after the test were recorded.

### 2.9 Western blotting

Rats were sacrificed by decapitation after the behavioral tests. Whole brains were rapidly removed from rats, and hippocampus was dissected on a cold plate. The hippocampus samples were then homogenized with ice‐cold RIPA buffer containing protease and phosphatase inhibitors followed by centrifugation at 12,000 rpm for 8 min at 4°C. The resulting supernatant was collected, and total protein concentration was determined in that using the bicinchoninic acid (BCA) method (Thermo, USA). The protein lysate (40 μg) was loaded on SDS‐polyacrylamide gel electrophoresis and transferred to polyvinylidene difluoride membranes. After blocking with 5% BSA for 1 h, the membrane was incubated with the following antibodies in TBST containing 5% BSA at 4°C overnight: anti‐NMDAR2A (1:1000), anti‐NMDAR2B (1:1000), anti‐GAPDH (1:10,000) (all from Abcam Inc., USA). After incubation, the membranes were washed with TBST and were then incubated with a horseradish peroxidase‐conjugated secondary antibody (Boster; 1:2000) to detect the protein and visualized with electrochemiluminescence reagent. Image J software (National Institutes of Health, USA) was used to analyze the intensity of bands for quantification, and the results were reported as relative density to GAPDH.

### 2.10 Statistical analysis

All data were expressed as mean ± standard error of the mean (SEM). SPSS17.0 software was used for statistical analysis, and two‐tailed unpaired Student's *t*‐test was used to compare two groups. Electrophysiological data was analyzed using Clampfit 10.6 and GraphPad 8 was used for drawing. *p* < 0.05 was considered statistically significant.

## 3 RESULTS

### 3.1 Long‐term spaceflight composite stress induces depressive behaviors

**FIGURE 1:**
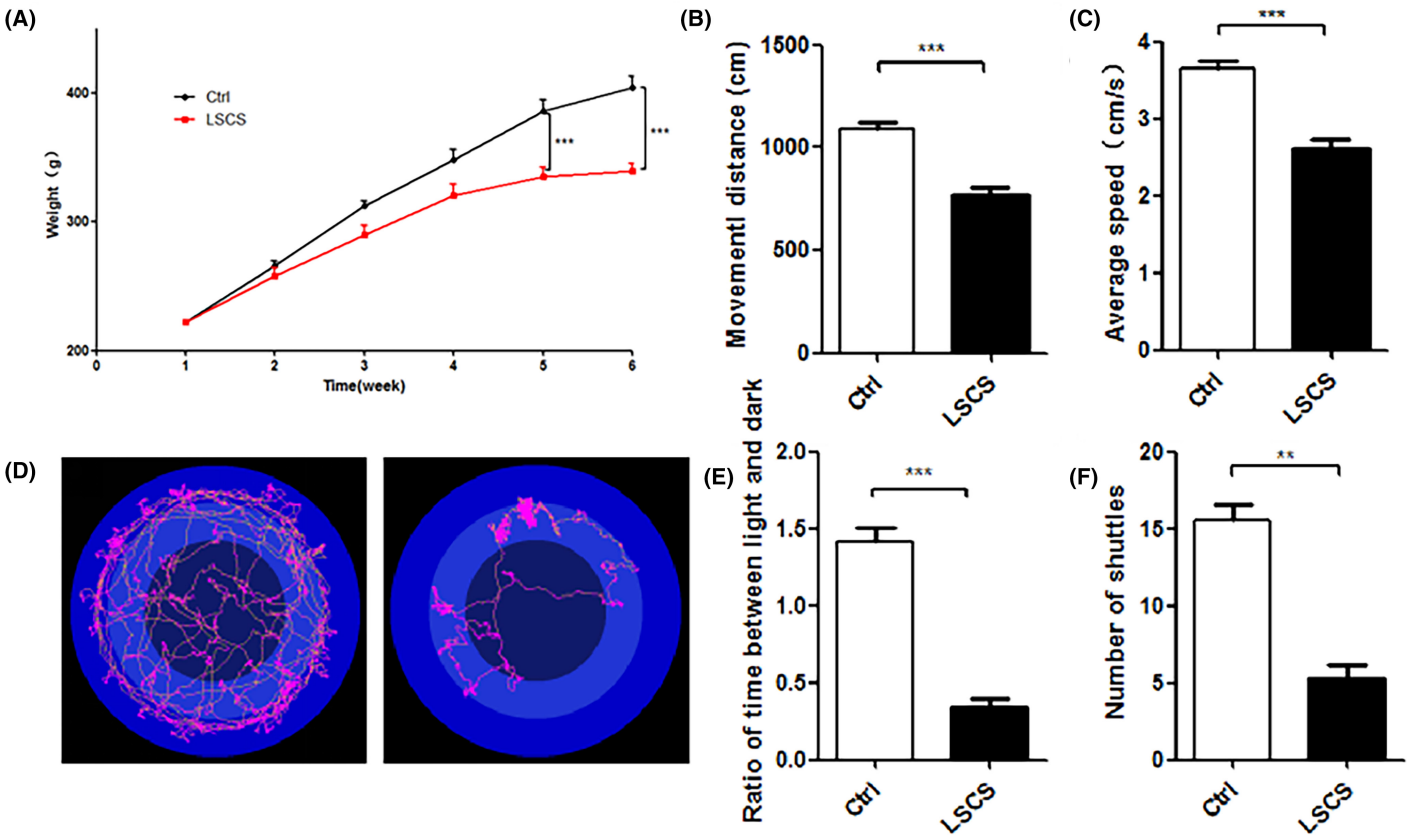
Long‐term spaceflight composite stress‐induced depressive behaviors. (A) Changes in body weight of rats during 6 weeks. (B, C) Effect of LSCS on movement distance and average speed in the open field test. (D) Typical diagram of the Ctrl and the LSCS group rat autonomous activity trajectory in open field. (E, F) Effect of LSCS on the number and time of light–dark box shuttle in rats. Data were expressed as means ± SEM (*n* = 10 for each group, **p < 0.01; ***p < 0.001).

To evaluate the potential role of LSCS in inducing depression, body weight measurement, open field test, and light–dark box shuttle test were performed to assess the depressive behaviors of rats. As shown in Figure 1A, weight changes during 6 weeks revealed that, compared with the Ctrl group, the weight of the LSCS group increased more slowly, and was significantly lower than the Ctrl group at the fifth and sixth weeks (*p* < 0.001; *p* < 0.001, respectively).

The open field test and light–dark box shuttle test were used to evaluate the level of anxiety and depression in rats exposed to LSCS. In the open field test, the movement distance and the average speed of the LSCS group were both dramatically lower when compared with the Ctrl group (*p* < 0.001; *p* < 0.001, respectively, Figure 1B–D). As to the light–dark box shuttle test, rats in the LSCS group obviously spent longer time in the dark box and conducted fewer shuttles compared to the Ctrl group (*p* < 0.001; *p* < 0.01, respectively, Figure 1E,F). These results suggested that exposure to LSCS promoted decreasing appetite and weight, depressive behaviors, and decreasing autonomous activity and interest in exploration in rats.

### 3.2 Long‐term spaceflight composite stress induces synaptic damage in hippocampus CA1 region

**FIGURE 2:**
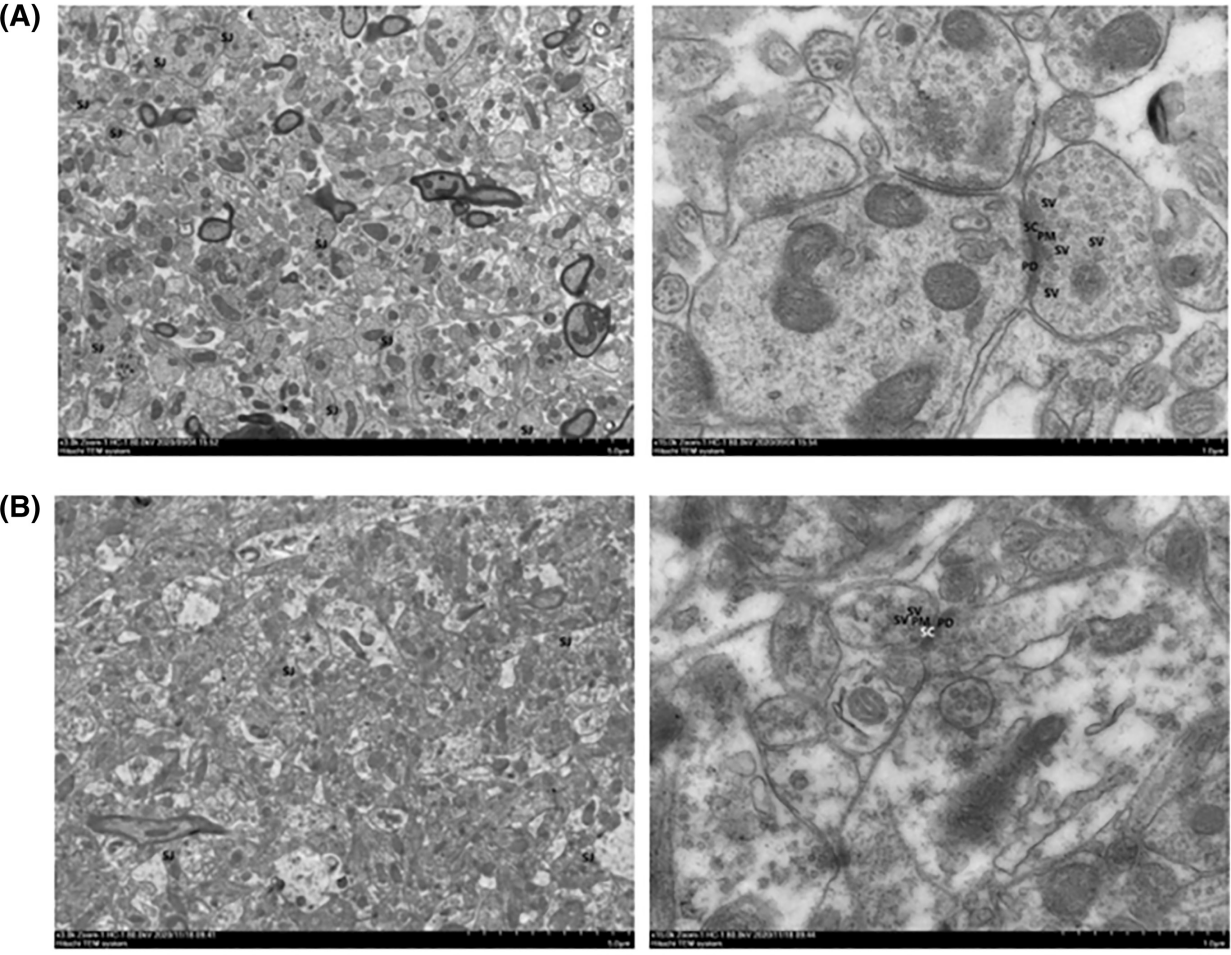
Long‐term spaceflight composite stress‐induced damage of synapse ultrastructure of the Ctrl (A) and the LSCS group (B). (The same field of view, the left side is magnified 3000 times; the right side is magnified 15,000 times).

To investigate the influence of LSCS on the synapse morphology, TEM was conducted to compare the synapse ultrastructure between the two groups. As shown in Figure 2A, the synapses in the Ctrl group was abundant and normal, the presynaptic membrane dense area (PM) was uniform and continuous, the postsynaptic membrane dense area (PD) was relatively thick, and the synaptic cleft (SC) was narrow. Furthermore, the synaptic vesicles (SV) were abundant and complete. While the structure was destroyed by LSCS (Figure 2B), demonstrated by less synapses, swelling of the synaptic body, thinning of pre‐ and post‐synaptic membrane dense areas, reduced electron density, widening of the synaptic cleft, and irregular or damaged synaptic vesicles.

We further measured the synaptic interface structural parameters to explore specific changes in synaptic ultrastructure. It is worth noting that compared with the Ctrl group, the PSD thickness and the synaptic interface curvature of LSCS group both significantly reduced (*p* < 0.05; *p* < 0.05, respectively), and the length of synaptic active area also slightly decreased, as shown in Table 1. These findings suggested that the abnormal morphology and structure of hippocampus synapses in the LSCS group was observed under TEM, indicating that LSCS could induce obvious damage in synapse structures, and thus lead to impaired synaptic structural plasticity.

**TABLE 1:** Structural parameters of synaptic interface (*n* = 10 for each group, *p < 0.05).

Group	PSD thickness (nm)	Length of synaptic active area (nm)	Synaptic interface curvature
Ctrl	47.63 ± 5.17	400.09 ± 61.32	1.091 ± 0.0211
LSCS	32.63 ± 1.33*	319.42 ± 22.42	1.035 ± 0.0086*

### 3.3 Long‐term spaceflight composite stress disrupts synaptic plasticity through decreasing LTP and inhibiting NMDA receptor channel currents

**FIGURE 3:**
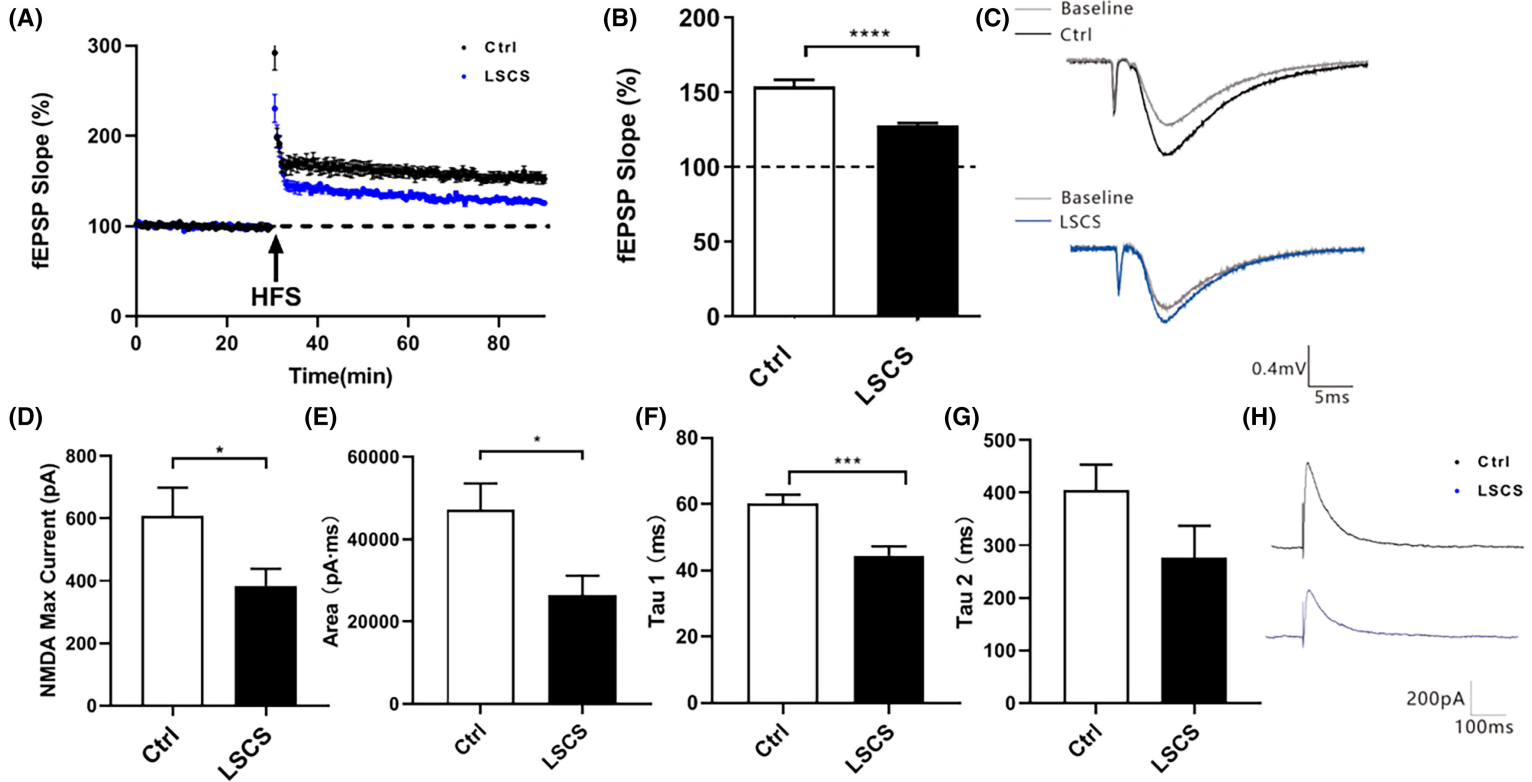
Long‐term spaceflight composite stress disrupted synaptic plasticity through inhibiting NMDAR channel currents. (A, B) Records of LTP in the hippocampus CA3‐CA1 region of rats in the Ctrl and the LSCS group (Ctrl 153.96% ± 4.52%, LSCS 127.99% ± 1.39%, *n* = 9 slices from three mice for each group). (C) Typical diagram of LTP of the Ctrl and the LSCS group. (D–G) The effect of LSCS on the maximum current (D) and maximum current area (E) of NMDAR channel currents in rat hippocampus CA1 region. The effect of LSCS on inactivation time constants of the two subtypes NR2A (F) and NR2B (G) (*n* = 9 slices from three mice for each group). (H) Typical diagram of NMDAR currents of the Ctrl and the LSCS group. Data were expressed as means ± SEM (*p < 0.05; ***p < 0.001; ****p < 0.0001).

To investigate the underlying mechanisms of LSCS on the possible damage of synaptic functional plasticity in the hippocampus, we performed patch‐clamp to record the LTP in the hippocampus CA3‐CA1 region to evaluate synaptic plasticity. As shown in Figure 3A, induced by HFS at 100 Hz, the fEPSP amplitude of the rats in the Ctrl group has exhibited a noticeable increase exceeding 50% over the baseline, indicating that the LTP was successfully induced. Compared to the Ctrl group, the fEPSP in the hippocampus CA3‐CA1 region of the LSCS group was significantly decreased (*p* < 0.0001, Figure 3B). These findings suggested a profound impairment of synaptic plasticity in this particular region.

In order to further explicate the mechanism for the observed decrease in LTP, precise measurements of NMDA receptor (NMDAR) channel currents were conducted, given its close association with the facilitation of LTP and synaptic plasticity. We found that as shown in Figure 3D,E, compared with the Ctrl group, the maximum current and maximum current area of NMDAR channel current were both significantly decreased in the LSCS group (*p* < 0.05; *p* < 0.05, respectively). We further measured the inactivation time constants of the two subtypes of NMDAR NR2 subunit‐NR2A and NR2B to explore whether the reduction in NMDAR channel currents was related to them. Intriguingly, the inactivation time of the NR2A subtype was significantly shortened in the LSCS group (*p* < 0.0001, Figure 3F), indicating that its inactivation rate was significantly increased. The inactivation time of the NR2B subtype also showed a decreasing trend (Figure 3G). The above results suggested that LSCS may inhibit NMDAR channel currents by inactivating the two major subtypes of NMDAR, thereby inhibiting the normal open of the NMDAR channel.

### 3.4 Long‐term spaceflight composite stress affects the expression of NMDAR channel proteins

**FIGURE 4:**
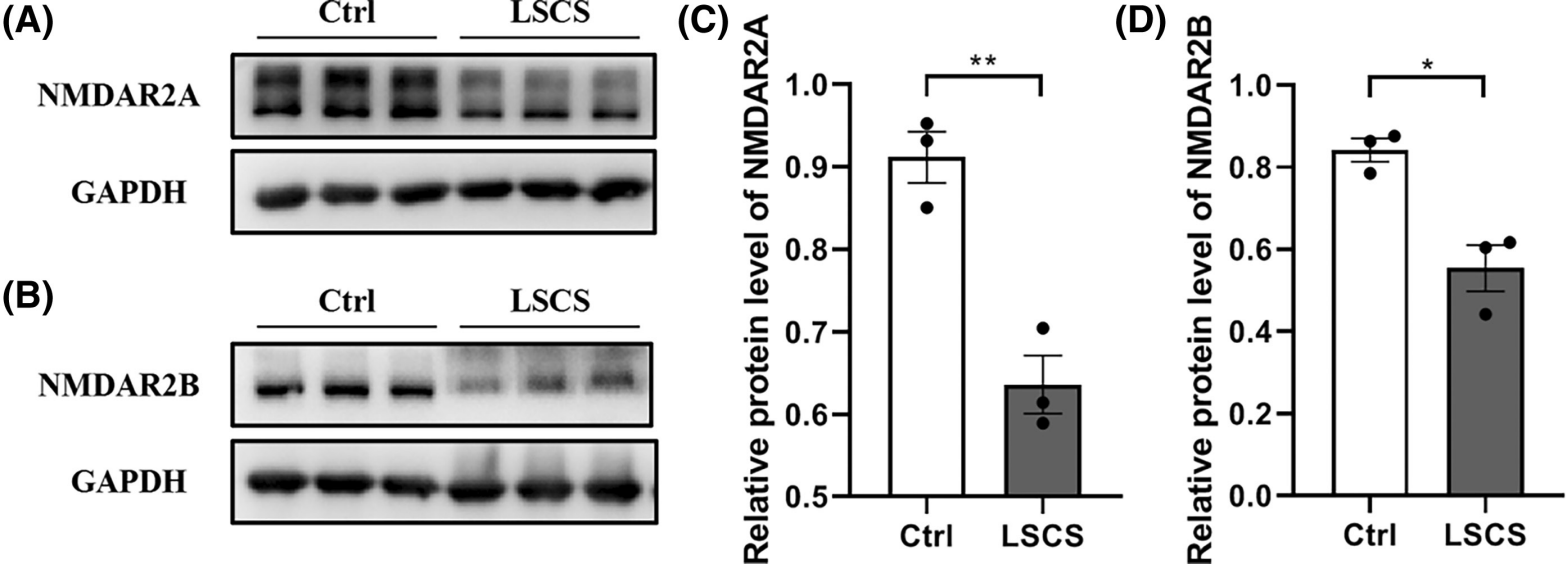
Long‐term Spaceflight Composite Stress affected the expression of NMDAR channel proteins. (A, B) Representative western blotting analysis of NMDAR2A and NMDAR2B expression in the Ctrl and the LSCS group (*n* = 3 for each group, Appendix S1). (C, D) Quantification of NMDAR2A and NMDAR2B protein levels of (A, B). Data were expressed as means ± SEM (*p < 0.05; **p < 0.01).

To validate the impact of LSCS on the subtype proteins NMDAR2A and NMDAR2B, the protein expression levels of the two groups were evaluated and compared. As shown in Figure 4A–D, western blotting revealed that the protein level of NR2A and NR2B was both significantly decreased in the LSCS group (*p* < 0.01; *p* < 0.05, respectively). The outcome indicated that LSCS appears to impede NMDAR channel currents by reducing the levels of NR2A and NR2B expression.

## 4 DISCUSSION

In this study, we investigated the underlying molecular mechanisms of depressive behavior in rats after 42 days of LSCS exposure. For the first time, we constructed the LSCS model via five factors, including microgravity, isolation, noise, circadian rhythm disruptions, and low pressure, to simulate the environment of real space station and invest possible causes of depression in astronauts. Behavioral experiments were then used to assess the influence of LSCS exposure on emotional behavior in rats. The open‐field experiment mainly judges the cognitive ability, tension, and interest of rats by testing the changes in their motor behaviors, which is a classic behavioral experiment to evaluate the spontaneous activity and anxiety state of the animals.^15^ The light–dark box shuttle experiment is based on the behavioral changes of rodents caused by the conflict between the innate aversion to strong light and the spontaneous exploration trend to novel environments.^16^ The ratio of box residence time is used to evaluate the anxiety and depression degree. In this experiment, LSCS reduced the speed and distance of the rats' movement in the open‐field experiment, as well as the times of shuttle and the time to explore the bright areas in the light–dark box shuttle experiment. Moreover, rats in the LSCS group all showed weight loss and obvious depressive behaviors. Thus, we have confirmed that LSCS could induce the symptoms of depression in rats.

Synapses are the basic structures for information transmission and processing between neurons.^17^ Changes in synaptic connections, often referred to synaptic plasticity, including synaptic structural plasticity and synaptic functional plasticity.^18^ Synaptic plasticity is very important for individuals to maintain normal function in ever‐changing internal and external environments, which enables brain to produce adaptive behaviors.^19^ However, abnormal synaptic plasticity is often associated with neurological diseases.^20^ Recently, the relationship between depressive behavior and synaptic plasticity in normal situations has been demonstrated repeatedly.^21^ Given these evidences, we decided to explore the possible causes in terms of synaptic plasticity under LSCS. Here, TEM was used to observe synaptic morphology in the hippocampus CA1 region. Results showed that synaptic morphology was severely impaired in the LSCS group, which may contribute to a reduction in synaptic structural plasticity. We further measured the synaptic ultrastructural parameters, including PSD thickness, length of synaptic active area, and synaptic interface curvature. Among them, PSD is a macromolecular complex that contains neurotransmitter receptors (e.g., glutamate receptors), cytoskeletal proteins (e.g., PSD‐95), and other proteins that are the key morphological basis of synaptic plasticity.^22^, ^23^, ^24^ The length of the synaptic active area is a basic index to the size of the synaptic contact area and is closely related to the timeliness of synaptic exocytosis.^25^ Furthermore, synaptic interface curvature is another important parameter of synaptic structural plasticity: a larger synaptic curvature can expand the contact area between the postsynaptic membrane and neurotransmitters, thereby reducing the spread of the neurotransmitter to the surrounding areas and improving the effectiveness of neural information transmission.^26^ An interesting observation of our study was that both PSD thickness and synaptic interface curvature have decreased significantly under LSCS, which may lead to ineffective information transmission between synapses, thereby impairing synaptic plasticity.

In terms of synaptic plasticity, LTP should be one of the best‐studied forms, which is a typical electrophysiological index for learning and memory functions, and can accurately reflect whether the synaptic plasticity is normal or not.^27^ We, therefore, adopted patch‐clamp to measure the LTP in the hippocampus CA3–CA1 region of the two groups. As expected, LTP has shown an obvious reduction in the LSCS group. As we know, LTP occurs in many pathways of the brain, and the most prominent form is induced following activation of the NMDARs.^28^, ^29^ The induction of LTP requires HFS to depolarize the postsynaptic membrane, then activate the NMDARs, open calcium ion channels to allow Ca^2+^ influx, and finally trigger LTP.^30^ The NMDAR channel is highly permeable to Ca^2+^ and is one of the main intracellular calcium ion channels.^31^, ^32^ It can be speculated that the open and close status of NMDAR channels would directly affect the induction of LTP. Thus, we measured the NMDAR channel currents. The results verified our conjecture that the maximum current value and maximum current area of NMDAR channels has both decreased, indicating that LSCS inhibited the NMDAR channels and even shut some of them down. The subsequent results could be the inability of extracellular calcium ions to flow into smoothly, and thereby failure of inducing LTP. This crucial calcium channel protein is composed of seven subunits (NR1, NR2A‐D, NR3A‐B), which is a tetrameric complex whose functional properties are tightly linked to the two subtypes‐NR2A and NR2B.^33^ We then investigated the inactivation time constants of NR2A and NR2B that positively correlated with channel opening. The smaller the inactivation time constant is, the faster the rate of inactivation will be, and vice versa. We found that LSCS obviously shortened the inactivation time of the NR2A subtype, and the NR2B subtype also showed a trend of shortening. This suggested that LSCS likely disrupted NMDAR channel opening by accelerating the inactivation of the two major subtypes of NMDAR. To prove our suspect, we measured the protein expression of the two subtypes. Results confirmed that the expression of both proteins was significantly decreased, which was consistent with our presumed results.

There are several limitations to our current study. First, the sex of the experiment animals should be taken into account. Due to the fact that around ninety percent of astronauts are male, we chose male rats as our subjects. However, the number of female astronauts is growing rapidly, it is insufficient to only evaluate the influence of LSCS on male. In addition, the age of experimental animals should also be more diverse to draw more comprehensive conclusions.

Collectively, we have discovered that exposure to LSCS would induce depressive behaviors in rats due to impaired synaptic plasticity in the hippocampus. In addition, the underlying molecular mechanism could be that LSCS lead the failure of NMDAR channels to open normally by inhibiting the expression of key channel proteins NR2A and NR2B, resulting in the dysregulation of intracellular calcium levels, which prevents LTP from being triggered normally, and ultimately leads to impaired synaptic plasticity. This work has provided a possible molecular explanation for synaptic plasticity dysfunction in relation to symptoms of depression under LSCS and important implications for the development of more effective and targeted treatments for symptoms of depression in the future of space medicine.

## AUTHOR CONTRIBUTIONS

Yi‐Shu Yin designed and performed the majority of the laboratory work. Yuan‐Bing Zhu, Jun‐Lian Liu and Quan‐Chun Fan assisted in the behavioral test. Shuang Zhao and Xiao‐Rui Wu helped get animal tissue. Jia‐Ping Wang and Yu Liu helped do the data analysis. Yong‐Zhi Li and Wei‐Hong Lu revised the manuscript.

## FUNDING INFORMATION

This work was supported by 1226 Major Project (AWS16J018).

## CONFLICT OF INTEREST STATEMENT

The authors declare that they have no conflicts of interest.

## Supporting information


AppendixS1

## Data Availability

The data that support the findings of this study are available from the corresponding author upon reasonable request.
