# Supplementary material for: Long‐term spaceflight composite stress induces depressive behaviors in model rats through disrupting hippocampus synaptic plasticity
Source: CNS Neurosci Ther. 2023 Oct 17;30(3):e14438. doi: 10.1111/cns.14438 (PMC10916436; doi:10.1111/cns.14438)
Supplement: Supplementary file 1 — AppendixS1 [file CNS-30-e14438-s001.pdf]

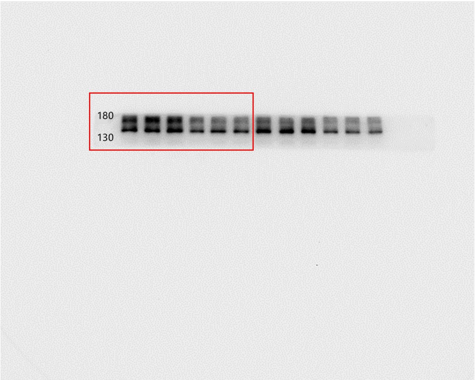

Full unedited gel/blot for Figure 4A-NMDAR2A

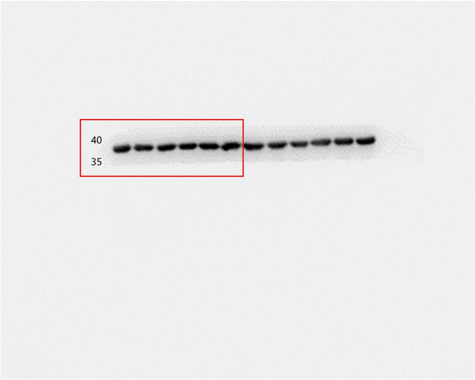

Full unedited gel/blot for Figure 4A-GAPDH

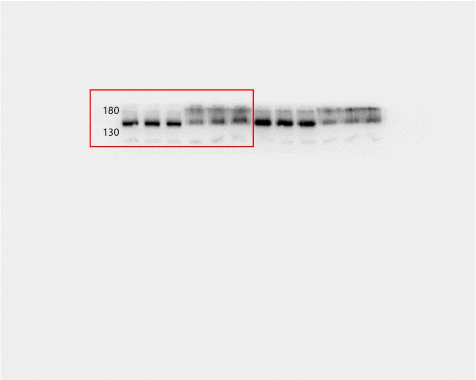

Full unedited gel/blot for Figure 4B-NMDAR2B

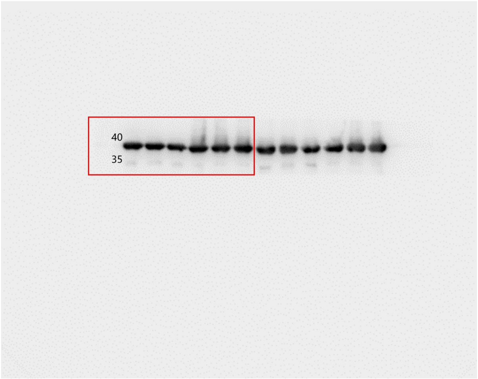

Full unedited gel/blot for Figure 4B-GAPDH
